# Feasibility of laparoscopic microwave ablation of caudate tumors: a case series

**DOI:** 10.1093/jscr/rjad478

**Published:** 2023-08-23

**Authors:** McKenzie J White, Alexander T Nelson, Jillian Wothe, Jacob S Ankeny, David Brauer, Christopher J Larocca, Eric H Jensen

**Affiliations:** Department of Surgery, University of Minnesota, 420 Delaware St SE, MMC 195, Minneapolis, MN 55455, United States; University of Minnesota Medical School, 420 Delaware St SE, MMC 195, Minneapolis, MN 55455, United States; Department of Surgery, Brigham and Women’s Hospital, 75 Francis St, Boston, MA 02115, United States; Department of Surgery, University of Minnesota, 420 Delaware St SE, MMC 195, Minneapolis, MN 55455, United States; Masonic Cancer Center, University of Minnesota, 420 Delaware St SE, MMC 806, Minneapolis, MN 55455, United States; Department of Surgery, University of Minnesota, 420 Delaware St SE, MMC 195, Minneapolis, MN 55455, United States; Masonic Cancer Center, University of Minnesota, 420 Delaware St SE, MMC 806, Minneapolis, MN 55455, United States; Department of Surgery, University of Minnesota, 420 Delaware St SE, MMC 195, Minneapolis, MN 55455, United States; Masonic Cancer Center, University of Minnesota, 420 Delaware St SE, MMC 806, Minneapolis, MN 55455, United States; Department of Surgery, University of Minnesota, 420 Delaware St SE, MMC 195, Minneapolis, MN 55455, United States; Masonic Cancer Center, University of Minnesota, 420 Delaware St SE, MMC 806, Minneapolis, MN 55455, United States

**Keywords:** caudate lobe of liver, microwave ablation laparoscopic colorectal liver metastases liver lesions

## Abstract

Microwave ablation of liver tumors allows preservation of liver parenchyma with good oncologic outcomes. However, ablation of tumors in the caudate lobe is particularly challenging. Adjacent critical anatomy, particularly the biliary hilum, has led to caudate location being considered a relative contraindication to ablation. To date, no series have described laparoscopic microwave ablation of caudate tumors of the liver. We describe our early experience with laparoscopic microwave ablation of caudate tumors. In this retrospective review of a prospectively maintained single-institution database, six patients with six primary or secondary caudate tumors underwent laparoscopic microwave ablation with no complications. At a median follow-up of 10.5 months, five out of six patients are free of caudate recurrence. Laparoscopic microwave ablation of caudate tumors is feasible. Long-term follow-up is needed to determine if local recurrence risk is higher than in other anatomical segments.

## INTRODUCTION

Microwave ablation is a widely utilized technique for treatment of primary and secondary tumors of the liver, but its utility may be limited by anatomic considerations or tumor location adjacent to heat sensitive critical structures. For this reason, tumor location in the caudate lobe has been considered a relative contraindication to thermal ablation [1]. This is because of the complex vascular anatomy of the area and its proximity to the biliary hilum [2]. Thermal injury to the central bile ducts can lead to catastrophic biliary stricture and even death [3]. As a result, ablation of tumors in the caudate lobe is seldom performed.

While percutaneous ablation of liver tumors is commonly performed, some anatomical segments are challenging to reach via that approach. Segments VII and IVa are difficult to target because of their posterior location and proximity to the heart, whereas subcapsular lesions may be at a higher risk of tumor rupture during ablation [4–6]. Superficial lesions may also be adjacent to heat sensitive organs, which must be dissected away prior to ablation. Lesions in the caudate that are at risk for thermal injury to the biliary tree require complex maneuvers to mitigate thermal spread. Some centers have reported the use of hydro-dissection to create space between the biliary tree and the area undergoing ablation [7–10]. Others have used nasobiliary tubes to cool the biliary tree while performing percutaneous ablation [11–13]. Despite these maneuvers, the caudate remains a technically difficult area to treat with percutaneous ablation [14].

Compared with percutaneous ablation, surgical ablation has several advantages [15]. Some authors have suggested that there is a lower recurrence risk with surgical, rather than percutaneous ablation, perhaps as a result of more aggressive surgical burns, or the ability to identify additional lesions during laparoscopy in a substantial number of patients [16]. Additionally, there are distinct technical advantages to the surgical approach. The liver, adjacent organs, and critical structures can be surgically mobilized to perform ablations safely. For instance, the porta can be retracted away from the area of caudate ablation to limit thermal spread, and access to the caudate is relatively simple from a surgical approach—it can be accessed by elevating the left lobe of the liver. Additionally, laparoscopic approach allows for easier placement of multiple MWA probes [17].

Recently, some groups have described thermal ablation of tumors in the caudate lobe using percutaneous radiofrequency and microwave techniques [18–20]. To date, no groups have described an operative approach to microwave ablation of tumors in the caudate lobe of the liver. Here we present a case series of patients who underwent laparoscopic assisted microwave ablation of the caudate lobe, describe our technique, and detail short-term outcomes.

## METHODS

### Patients

Patients who underwent laparoscopic microwave ablation of primary or secondary tumors of the caudate lobe of the liver between 2021 and 2022 were identified from a prospectively maintained single-institution database. For all patients, cross-sectional imaging with contrast-enhanced magnetic resonance imaging (MRI) was obtained pre- and post-ablation, with the goal of an ablated area 2 cm larger than the targeted tumor [21]. Ablations were performed laparoscopically under general anesthesia and were carried out by two experienced hepatobiliary surgeons (E.H.J. and J.S.A.).

### Surgical technique

**Figure 1:**
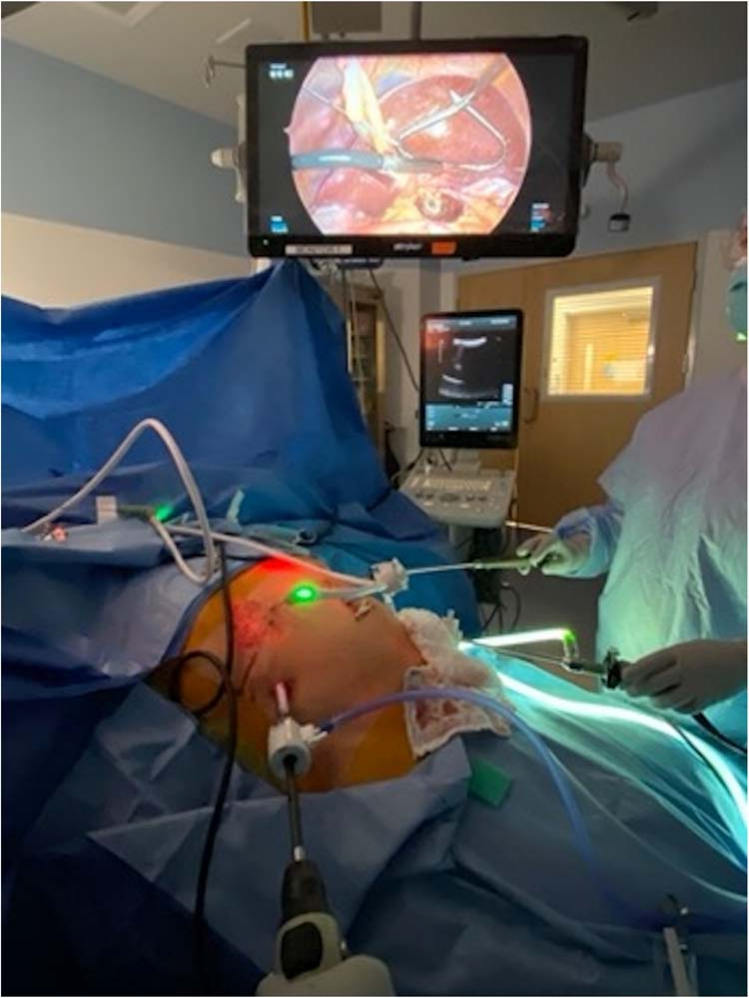
Patient positioned in reverse Trendelenburg to facilitate exposure of upper abdomen. Port placement: 12 mm port in right upper quadrant to facilitate laparoscopic ultrasound of liver, other 5 mm ports placed as needed for mobilization of liver. Laparoscopic image above patient demonstrates left lobe of liver elevated with retractor, laparoscopic ultrasound probe evaluating left lobe of the liver, and burned area visible in caudate lobe below.

**Figure 2:**
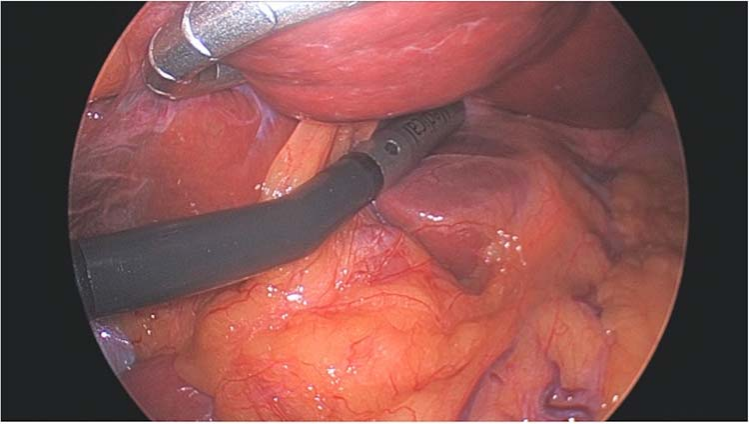
Left lobe of liver elevated with flexible liver retractor, facilitating laparoscopic ultrasound evaluation of caudate lobe.

All cases were performed under general anesthesia. Patients were positioned supine, with a 5 mm port placed at the umbilicus and a 12 mm port placed in right flank to allow for ultrasound of liver (Fig. 1). Additional 5 mm ports are positioned as needed for mobilization/retraction of the liver. Using ultrasound guidance (Flex Focus 800, BK Ultrasound), lesions identified on preoperative MRI were identified intraoperatively. Caudate lesions were accessed by elevating the left lobe of the liver (Fig. 2). In some cases, the porta hepatis was encircled with umbilical tape and retracted away from the ablation site. Ablations were carried out with percutaneous introduction of one or two microwave ablation probe(s) (2.45 GHz, PR15XT probes, NeuWave Microwave Ablation System, Ethicon). Ablations of the caudate lobe were performed either alone or in conjunction with ablation of lesions in other hepatic segments. One ablation was performed in combination with resection of the primary tumor, which was of colonic origin.

### Outcomes

Outcomes measured included complications, hospital length of stay, and caudate recurrence. Complications were defined as any intraoperative or postoperative event that necessitated medical or surgical intervention. Length of stay was measured in days from procedure. Follow-up was calculated from date of ablation to date of most recent imaging. Local recurrence was defined as any radiographic evidence of tumor adjacent to a previous burn site at any time. Primary treatment failure was defined as persistent enhancement of tumor on post-procedure imaging. Time to recurrence was calculated from date of ablation to date of imaging demonstrating recurrence.

### Statistical analyses

Descriptive statistics were used to describe the outcomes listed above. This research was approved by the University of Minnesota Institutional Review Board (Study 00003995).

## RESULTS AND CASE SERIES

**Figure 3:**
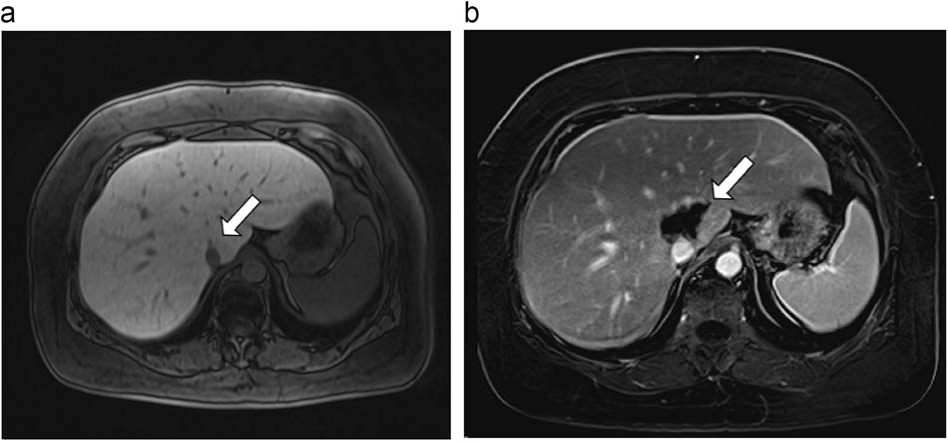
(**a**) Pre-ablation MRI demonstrates 6 mm lesion in caudate lobe of liver. (**b**) Post-ablation MRI (18 days postoperatively) demonstrates 40.4 mm complete ablation site in caudate lobe of liver.

**Figure 4:**
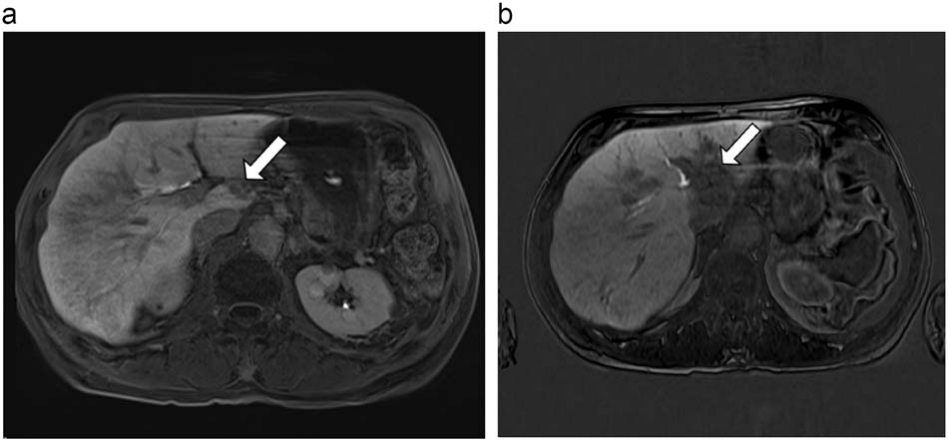
(**a**) Pre-ablation MRI demonstrating 15 mm lesion in caudate lobe of liver (image degraded by motion artifact). (**b**) Post-ablation MRI (20 days postoperatively) demonstrating 35 mm complete ablation of caudate lesion (image degraded by motion artifact).

**Figure 5:**
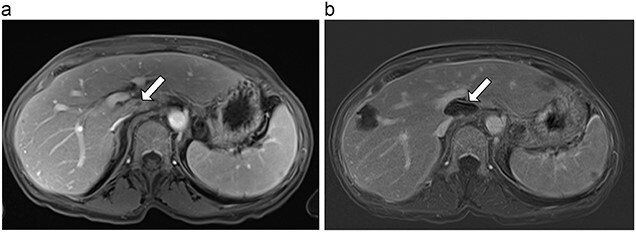
(**a**) Pre-ablation MRI demonstrating 5 mm lesion in caudate lobe of liver. (**b**) Post-ablation MRI (19 days postoperatively) demonstrating 36.2 mm complete ablation of caudate lesion.

**Figure 6:**
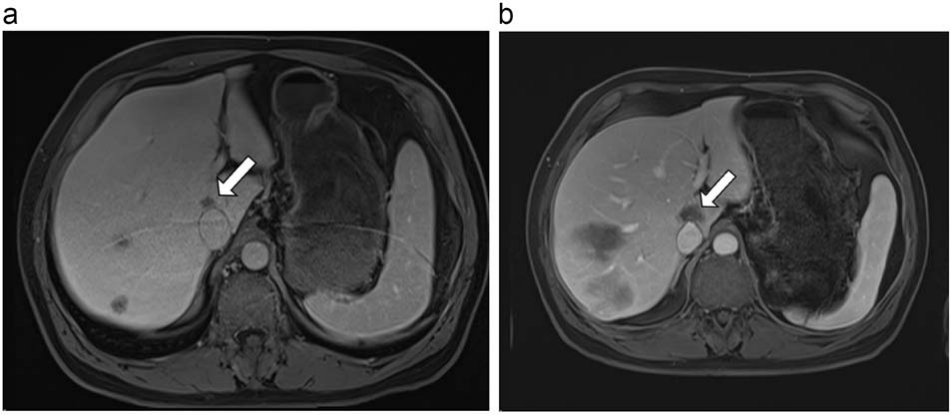
(**a**) Pre-ablation MRI demonstrating 14 mm lesion in caudate lobe of liver. (**b**) Post-ablation MRI (23 days postoperatively) demonstrating 23.8 mm complete ablation of caudate lesion.

**Figure 7:**
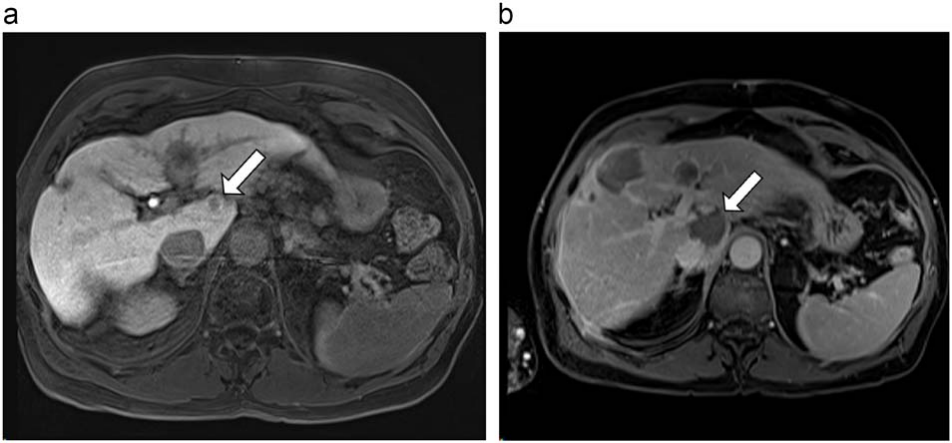
(**a**) Pre-ablation MRI demonstrating 10 mm caudate lesion. (**b**) Post-ablation MRI (14 days postoperatively) demonstrating 35.9 mm complete ablation of caudate lesion.

**Figure 8:**
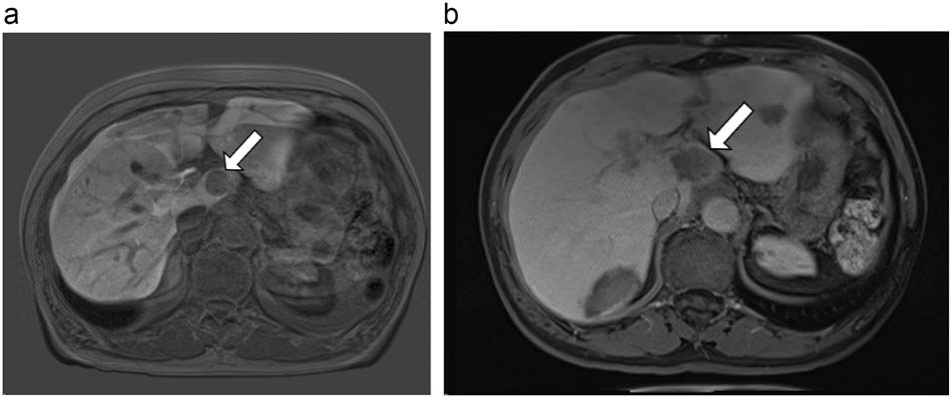
(**a**) Pre-ablation MRI demonstrating 28 mm caudate lesion. (**b**) Post-ablation MRI (16 days postoperatively) demonstrating 37.3 mm complete ablation of caudate lesion.

Six patients with six primary or secondary tumors of the caudate lobe were identified (Table 1). Median age at diagnosis was 68 years (51–77 years); two patients were female. Primary disease process was colon adenocarcinoma *(n* = 4; Figs 3–6), hepatocellular carcinoma (HCC; *n* = 1; Fig. 7) and metastatic pancreatic neuroendocrine tumor (*n* = 1; Fig. 8). All patients with colon adenocarcinoma received systemic therapy with 5-fluorouracil, oxaliplatin, and leucovorin (FOLFOX) prior to microwave ablation of hepatic metastases. The patient with metastatic neuroendocrine tumor had progression of hepatic metastases on lanreotide prior to microwave ablation. The patient with HCC had previously been treated with transarterial embolization and cryoablation of tumors, but had recurrence of disease, which was managed with microwave ablation.

**Table 1:** Summary of patient characteristics, disease characteristics, treatment details, and recurrence.

Patient (figure)	Age, sex	Diagnosis	Therapies prior to ablation	Total number of lesions ablated	Procedure	Caudate lesion size (mm)	Caudate ablation size (mm)	Difference between ablation size and lesion size (mm)	Follow-up (months)	Caudate recurrence
1 (3a,b)	61, F	Colon adenocarcinoma	6-month FOLFOX	1	Laparoscopic microwave ablation	6	40.4	34.4	10	No
2 (4a,b)	67, M	Colon adenocarcinoma	8 cycles capecitabine and oxaliplatin	6	Laparoscopic microwave ablation	15	35	20	6	No
3 (5a,b)	68, F	Colon adenocarcinoma	6 cycles modified FOLFOX-6	8	Laparoscopic microwave ablation and low anterior resection of colon	5	36.2	31.2	15	No
4 (6a,b)	51, M	Colon adenocarcinoma	6 cycles FOLFOX	4	Laparoscopic microwave ablation	14	23.8	9.8	13	Yes
5 (7a,b)	71, M	HCC	Transarterial embolization and cryoablation of liver lesions	3	Laparoscopic microwave ablation	10	35.9	25.9	9	No
6 (8a,b)	77, M	Pancreatic neuroendocrine tumor	Lanreotide	19	Laparoscopic microwave ablation	28	37.3	9.3	10	No

A total of 41 lesions [19, 8, 6, 4, 3, and 1] were ablated in six patients with a single-caudate lesion ablated in each patient. Median diameter of caudate lesions was 12 mm (5–28 mm). Two lesions directly abutted the inferior vena cava. Ablations were performed laparoscopically in all cases. Ablations of the caudate were performed alone (*n* = 1) or in conjunction with ablation of lesions in other liver segments (*n* = 5). In one case, ablation was performed in conjunction with resection of the primary disease site (colon). Estimated blood loss was minimal (<30 ml per case). There were no complications related to the ablative procedure.

Of five patients who underwent ablation alone, four were discharged on the day of the procedure and one was hospitalized for 2 days postoperatively. This was done to monitor for tumor lysis syndrome, as the patient had undergone ablation of 19 lesions [22].

Postoperative MRIs were obtained at a median of 19 days postoperatively (14–23 days). Median ablation margin (difference between ablation zone and original tumor size) was 22.95 mm (9.3–34.4 mm). At a median follow-up of 10.5 months (6–15 months), one of six (17%) caudate ablations had evidence of local recurrence. At diagnosis, this tumor had been abutting 180 degrees of the vena cava, and its proximity to the posterior hilum made a more aggressive burn inadvisable. Time to recurrence for this patient was 6 months. There were no recurrences in non-caudate ablations in this same patient. No long-term complications were observed in any patients.

## DISCUSSION

In this single-institution case series, six patients underwent laparoscopic microwave ablation of six primary and secondary tumors of the caudate lobe of the liver. There were no postoperative complications and there was a single case of caudate recurrence.

Our group and others have previously described the use of laparoscopic microwave ablation for patients with colorectal liver metastases, and the durable outcomes of operative microwave ablation for this disease [21, 23]. Several groups have described percutaneous ablation of caudate lesions, but these have been limited to HCC [18–20, 24]. To our knowledge, there are no reports of laparoscopic microwave ablation for caudate tumors—either primary liver tumors such as HCC, or metastatic disease [15]. In this case series, we report the feasibility of laparoscopic microwave ablation of caudate tumors—either primary or secondary.

Laparoscopy provides the advantage of being able to retract the portal structures away from the ablation area, as described in this series. With this technique, there were no incidences of biliary tract injury. Additionally, via the surgical approach, all caudate lesions were easily visualized and accessed with mobilization of the left lobe of the liver. In all cases, there was good technical success of each ablation, with postoperative MRI demonstrating adequate ablation of caudate lesions. There were no instances of biliary stricture. Five of six patients are free of caudate recurrence at a median of 10.5 months from ablation.

The purpose of this study was to assess the feasibility and short-term outcomes of laparoscopic caudate ablation. Further studies will be required to define long-term caudate recurrence risk. In terms of technical limitations, it is likely that even with evidence from this series, tumor proximity <1 cm from the bile duct will remain a contraindication to ablation in order to avoid thermal injury to the central biliary tree [25]. Finally, we wish to caution that these procedures should not be attempted by inexperienced surgeons as they require technical expertise with laparoscopic liver surgery, intraoperative ultrasound, and ablation. Despite these limitations, this case series establishes the feasibility of laparoscopic microwave ablation for caudate tumors and may contribute to future studies evaluating long-term oncologic outcomes.

## CONCLUSION

This single-institution case series of patients who underwent laparoscopic microwave ablation of caudate liver lesions demonstrates both safety and short-term efficacy. Further studies will be required to evaluate long-term local recurrence risk, as this may be higher than in other anatomic segments.
